# Human neutrophil lipocalin (HNL) as a biomarker of acute infections

**DOI:** 10.1080/03009734.2017.1420112

**Published:** 2018-02-23

**Authors:** Per Venge

**Affiliations:** Department of Medical Sciences, Clinical Chemistry, Uppsala University, Uppsala, Sweden

**Keywords:** Clinical chemistry, immunoassays, infectious diseases

## Abstract

The early and accurate discrimination between bacterial and viral causes of acute infections is the key to a better use of antibiotics and will help slow down the fast-growing resistance to commonly used antibiotics. This discrimination is in the vast majority of cases possible to achieve by blood assay of the biomarker human neutrophil lipocalin (HNL), which we showed to be uniquely increased in patients suffering from bacterial infections. In serum, sensitivities and specificities of >90% are achieved in both adults and children. In order to eliminate the need to produce serum, a whole-blood assay with an assay time of <10 min was developed in which blood neutrophils are activated to release HNL. The diagnostic accuracy of this assay also showed sensitivities and specificities of >90% in most infectious diseases and was clearly superior to contemporary assays such as blood neutrophil counts, C-reactive protein, procalcitonin, and expression of CD64 on blood neutrophils. This format lends itself to the development of a point-of-care HNL assay and will be a major step forward to accomplish the goal of accurately diagnosing patients with symptoms of acute infections within 10 min at the emergency room or at the doctor’s office.

## Introduction

Acute infections, either caused by virus or bacteria, affect almost anyone at least once a year. Most of these infections are harmless and will clear up after a couple of days without any further treatment. However, in many cases the infection stays or causes symptoms that make the subject call for medical advice and hopefully treatment. The vast majority of such patients will visit the primary care doctor, and the appropriate and common question asked by the doctor should be: ‘Should my patient be prescribed antibiotics?’ This question is also one of the most important questions asked in medical care, since the unnecessary prescription of antibiotics increases the likelihood of further development of antibiotic resistance, a phenomenon that has turned into a serious threat to mankind (1–3). Thus, in subjects affected by common viral infections, and who are not regarded as risk patients or as particularly vulnerable, antibiotics are ineffective and should be avoided—and the answer to the question should be ‘No’.

How will the doctor know when to treat with antibiotics and when to avoid them? The first judgment is based on the medical history and physical examination of the patient. However, the signs and symptoms of many acute infections either caused by virus or bacteria are very unspecific and do not allow for an accurate distinction to be made. Previous studies have shown that when experienced physicians try to distinguish between viral and bacterial causes of respiratory tract infections there is a sensitivity and specificity of no more than 55%–60% when based on these criteria alone (4,5). The need for additional means to increase the diagnostic accuracy is therefore obvious, and traditionally white blood cell counts or differentials are used and have been shown to be very useful. In many countries these measures have been replaced by plasma protein measurements such as C-reactive protein (CRP). With these additions the sensitivity and specificity are increased and approach 70%–80%. These figures show that still a large proportion of our patients are falsely diagnosed with the consequence of maltreatment.

This overview will briefly summarize the current knowledge of diagnostic means of acute infections and show that the measurement of one protein, human neutrophil lipocalin (HNL), secreted from the activated neutrophils, seems to be superior in this regard. The emphasis will be on the early and immediate distinction between viral and bacterial causes of acute infections with a focus on ruling out those subjects in whom antibiotic treatment should not be prescribed. A negative predictive value (PV_neg_) of a test to be used to rule out a possible bacterial infection should ideally be 100%, but realistically a PV_neg_ exceeding 90% should be acceptable. The specific detection of the causing agents, i.e. all the important microbiology tests, will not be discussed. Such tests are important in the guidance of which antibiotics to use, but do not fulfill the need for rapid answers to the doctor’s immediate decision whether or not to prescribe antibiotics.

## Biochemical and cellular means to diagnose acute infections

There are a number of biochemical and cellular tests that are currently used or that have been proposed as diagnostic means of acute infections (Table 1). In the following I will discuss some of these based on our own experience. To be useful, the test should be easily available and results provided within a few hours. Ideally the preferred test should be provided as a point-of-care test, i.e. a test sufficiently robust to be run without the involvement of expert technicians and close to the care provider, e.g. in a doctor’s office. At this moment few of the listed tests fulfill these requirements.

**Table 1.** Biochemical and cellular diagnosis and monitoring of acute infections. Which are the possibilities?

• White blood cell counts and differentials • Acute phase proteins, e.g. CRP, SAA • Cytokines, e.g. IL-6, IL-8, G-CSF, TNF-α, IP-10 • Inflammatory cell markers, e.g. MPO, lactoferrin, HNL, lysozyme, HBP (azurocidin), soluble CEACAM8, α-defensins, calprotectin • Adhesion molecules, e.g. E-selectin, VCAM-1, ICAM-1 • Others, e.g. PCT, LBP, TREM-1, TRAIL • Cell surface markers, e.g. CD64, CD35, CD11b • Lactate

CD: cluster of differentiation; CEACAM: carcinoembryonic antigen-related cell adhesion molecule; CRP: C-reactive protein; G-CSF: granulocyte colony stimulating factor; HBP: heparin binding protein; HNL: human neutrophil lipocalin; ICAM: intercellular adhesion molecule; IL-6: interleukin 6; IL-8: interleukin 8; IP-10: interferon gamma-inducible protein 10; LBP: lipopolysaccharide binding protein; MPO: myeloperoxidase; PCT: procalcitonin; SAA: serum amyloid A; TNF-α: tissue necrosis factor-α; TRAIL: TNF-related apoptosis-inducing ligand; TREM: triggering receptor expressed on myeloid cells; VCAM: vascular cell adhesion molecule.

## HNL

Human neutrophil lipocalin (HNL) has also been called neutrophil gelatinase associated lipocalin (NGAL) or lipocalin 2 (Table 2). The names refer to the fact that HNL was first identified in and purified from human neutrophils (6–8). It is stored as a preformed molecule in the secondary (specific) granules of neutrophils and is readily released from this storage compartment upon activation of the cell. The production of HNL may also be induced in epithelial cells, of which the production by kidney epithelial cells has gained considerable interest lately, since this production is induced by processes affecting kidney function (9,10). Thus, in patients with acute kidney injury, increased concentrations of HNL were observed early on in the disease process. The true biological function of HNL is still not known, although it has been shown that HNL binds siderophores and has bacteriostatic properties. HNL also relates to apoptotic activities. Thus, infusion of HNL/NGAL in kidneys exposed to ischemia prevents kidney injuries (11).

**Table 2.** Human neutrophil lipocalin (HNL).

• Produced by human neutrophils and epithelial cells • Stored in secondary (specific) granules • Homology with other lipocalins • Function? Siderophore, mediates apoptosis? • Binds gelatinase B/MMP-9 • Monomer 24 kDa—homodimer 45 kDa • Also called NGAL (neutrophil gelatinase associated lipocalin) and lipocalin 2

MMP-9: matrix metalloproteinase-9.

HNL was first discovered in and purified from human neutrophils. We named the protein ‘human neutrophil lipocalin’, since the amino acid sequence showed a homology to other proteins of the lipocalin family of proteins. The major form purified from the neutrophils was that of a homodimer of a molecular mass of 45 kDa, but also monomeric (24 kDa) and heterodimeric (>90 kDa) molecular forms are present in neutrophils (7,12). The heterodimeric protein is a complex between HNL and gelatinase (MMP-9). Hence, the name neutrophil gelatinase associated lipocalin (NGAL) was given to the protein by a research group in Denmark (8). The homodimeric molecular forms seem unique to neutrophils, in contrast to epithelial cells, which only produce the monomeric form. This difference has been exploited by our group, since antibodies produced against the two molecular forms can be used in immunoassays to distinguish between HNL originating from neutrophils or epithelial cells (12,13). This distinction was shown to be of fundamental importance in the use of HNL measurements in various body fluids as a biomarker of neutrophil involvement in e.g. bacterial infections and kidney disease or as a biomarker of epithelial involvement in e.g. acute kidney injury.

Early after our discovery of HNL, we produced specific antibodies against the dimeric neutrophil-originating molecular form. This enabled us to establish a sensitive radioimmunoassay for the measurement of HNL in various body fluids. In serum/plasma the main contributor to the HNL concentrations is the population of activated neutrophils. Exceptions may be in patients with acute kidney injury (9,10) in whom contribution from kidney epithelial cells may occur and in patients with various advanced forms of cancer (14). Thus, in such diseases the assay in serum/plasma of HNL may be clinically useful if bacterial infections can be excluded. In otherwise healthy people, in whom the above diseases can be excluded, increased blood concentrations of HNL indicate the presence of a bacterial challenge.

## Serum HNL in adults with acute infections

**Figure 1.**
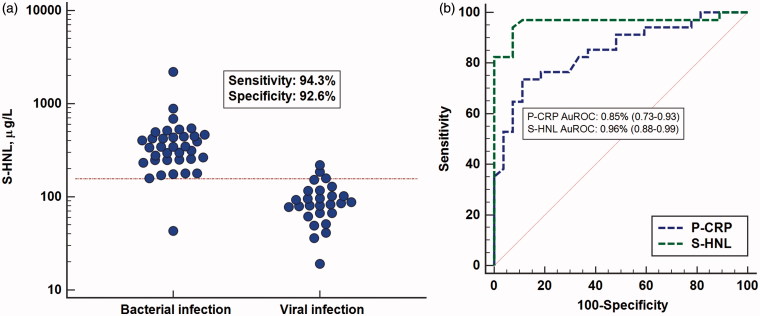
(a) Serum concentrations of HNL as measured by radioimmunoassay in patients with verified bacterial or viral cause of their acute infection. The horizontal line indicates +3 SD of the upper level of healthy non-infected subjects. Also shown in the figure are the sensitivity and specificity of HNL in the discrimination between bacterial and viral infections. (b) ROC curve analysis of the results of serum HNL and plasma CRP measurements shown in Figure 1a. The areas under the ROC curves (AuROC) are given in the figure.

Our first study on patients with acute infections included 140 patients admitted to the infection department at our hospital (15). Thirty-nine patients were excluded, since it could not be unequivocally determined whether the patient had a pure bacterial or a pure viral infection. Thus, the purpose of the study was to examine the serum concentrations in patients with a definite acute bacterial infection at admission and before antibiotic treatment and compare those to the HNL concentrations in a cohort with a definite acute viral infection. All clinical and laboratory means at hand were used for classifications of the patients. The concentrations of HNL in patients with bacterial infection were increased above normal concentrations (Figure 1(a); the horizontal line indicates mean +3 SD) and distinct from the concentrations in patients with acute viral disease. A ROC curve analysis indicated very high sensitivities and specificities, which were clearly superior to CRP (Figure 1(b)) and blood neutrophil counts. The positive predictive value (PV_pos_) and PV_neg_ in the distinction between the two causes of acute infections were 93% and 96%, respectively. Thus, the likelihood of having an acute bacterial infection with concentrations below 155 μg/L was very low. The potential of HNL being an acceptable rule-out assay of bacterial infections was accordingly very high.

In another study, HNL concentrations in serum were measured in patients with influenza A infection and compared to the concentrations in healthy subjects and in patients with bacterial infections without any signs of influenza A (16). Concentrations of HNL in serum of patients with influenza A were higher than those found in healthy subjects, but with some overlap. However, the concentrations were lower than those seen in patients with acute bacterial infections. It is also noteworthy that the concentrations in patients with bacterial infections, with one exception, had increased concentrations as compared to the healthy subjects. These results confirmed our previous study (15) that serum concentrations of HNL are powerful means to rule out bacterial causes of acute infections. Some of the influenza patients might have had secondary bacterial infections, a common but probably underdiagnosed consequence of influenza infections, which may explain some of the elevated HNL concentrations. An alternative explanation, which will be discussed further below, is the fact that some epithelial cells of the lungs may be induced to produce HNL. Thus, our early radioimmunoassay was not able to distinguish between dimeric and monomeric HNL, i.e. HNL originating from either neutrophils or epithelial cells

**Figure 2.**
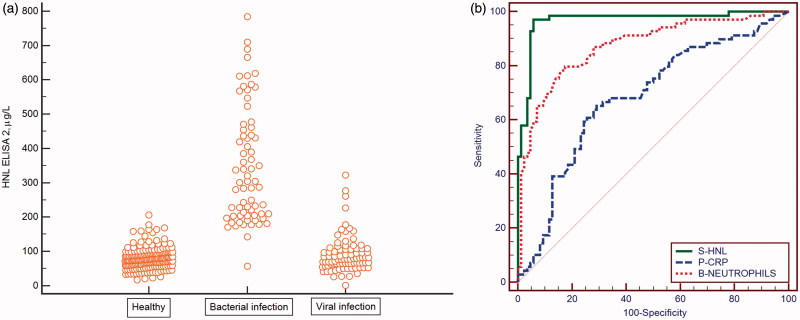
(a) Serum concentrations of dimeric HNL measured by our specific ELISA in patients with acute infections and in healthy non-infected controls. (b) ROC curve analysis of the results of serum HNL and plasma CRP and neutrophil counts in the patients shown in Figure 2(a).

In attempts to make our immunoassay more specific we developed a number of ELISAs based on the combination of several different monoclonal antibodies with different epitope specificities. In some of the assays we still took advantage of our polyclonal antibody produced against the dimeric HNL. In a study on about 350 patients with acute infections and non-infected controls the serum levels were measured by these different ELISAs (17). In this study it was clearly shown that some antibody combinations were superior to other combinations in the distinction between bacterial and viral infections. Thus, the combination of our polyclonal antibody with a monoclonal antibody directed against the dimeric HNL was superior in this distinction, whereas another ELISA in which a combination of antibodies directed towards all forms of HNL, i.e. both monomeric and dimeric, showed somewhat increased HNL concentrations in viral infections as compared to non-infected healthy controls and consequently a less clear distinction between the two causes of infections (Figure 2(a)). The optimal ELISA, i.e. the ELISA 2, showed a very high negative predictive value of 98.8% and a positive predictive value of 93.1%. Thus, the diagnostic performance of ELISA 2 was clearly superior to CRP and blood neutrophil counts (Figure 2(b)) (Table 3). These findings support our notion of above, when employing the radioimmunoassay for HNL measurements, that in some cases with viral infection, monomeric HNL is released to a greater extent, most probably from virally infected epithelial cells.

**Table 3.** The diagnostic specifications of S-HNL, P-CRP, and blood neutrophil counts in the discrimination between acute bacterial and viral infections.

Biomarker	AuROC % (95% CI)	Sensitivity	Specificity
S-HNL	0.97 (0.93–0.99)	97.1%	94.2%
P-CRP	0.68 (0.60–0.75)	65.2%	71.6%
B-neutrophils	0.87 (0.81–0.92)	79.7%	82.8%

## Serum HNL in children with acute infections

The accurate diagnosis of causes of acute infections in young children and newborns is difficult, since signs and symptoms are less obvious as compared to those seen in older children and adults. We conducted a study on a group of 87 newborns with suspected bacterial infections and found highly increased serum HNL concentrations in children with proven or suspected sepsis (18). The concentrations were in the range of those seen in adults with similar diagnoses. Also, concentrations in healthy neonates and children 3–5 days of age were similar to the concentrations of healthy adults. The difficult group was the children who presented with symptoms suggestive of bacterial infection, but in whom no infection could be proven. The most common clinical problems of these children were symptoms of respiratory distress. This cohort of children had concentrations very much in between normal concentrations and those of the septic children. In analogy with the findings in adults, it seems quite likely that the majority of these children actually were infected. The kinetics of HNL in these children showed the highest concentrations at day 0 in contrast to CRP, which peaked one day later. Normalization of HNL followed the clinical recovery of the children.

A study on older children was conducted in order to investigate the kinetics of serum HNL in comparison with other biomarkers such as CRP (19). The diagnosis of the cause of the acute infection was blinded and clinical, based on the signs and symptoms used in the day-to-day management of the children. As expected, HNL concentrations were increased in those patients deemed to have bacterial infections as opposed to those deemed to have a viral infection. The kinetics of serum concentrations of HNL after start of antibiotic treatment showed a significant reduction after one day and a normalization after two days of treatment in concert with the recovery of the children and presumed eradication of the bacterial challenge. This was clearly contrasted by findings with CRP that stayed elevated in the majority of children even at days 2 and 3. This is an important observation, since the use of CRP as a tool to diagnose bacterial infections may be grossly misleading late in the course of an infection. The lagging behind of CRP is a well-known phenomenon and has to be kept in mind when using CRP in the diagnosis of acute infections in order to prevent unnecessary antibiotic treatment in a subject who has already eradicated the bacterial challenge. Thus, the two studies on children show that the kinetics in rise and fall of HNL and CRP differ considerably, with an earlier rise of HNL as a consequence of the bacterial challenge and an earlier fall of HNL after antibiotic treatment as compared to CRP. In addition to the more accurate distinction between bacterial and viral infection, these kinetics should further aid in the better use of antibiotics.

## Serum HNL in other inflammatory diseases

An important issue is the specificity of HNL when it comes to the diagnosis of acute infections. As mentioned above, HNL in serum/plasma may originate from two major sources. One is determined by the turnover and activation of the neutrophils in the body and the other by *de novo* synthesis of HNL by epithelial cells in the kidney and by various forms of cancer cells. Activation of neutrophils in the body may occur in other inflammatory diseases, and in those diseases you expect elevated blood concentrations of HNL in the absence of any infection. This was, however, not the case in patients with rheumatoid arthritis in whom HNL concentrations were normal in spite of active clinical disease (20). In patients with cystic fibrosis, HNL accurately identified those with acute pulmonary exacerbations (21). Also in patients with chronic obstructive pulmonary disease (COPD), HNL was increased concomitant with exacerbations, which probably is a sign of the bacterial infection being a major cause of deterioration of these patients (22). HNL concentrations are slightly elevated by major surgical trauma, but the elevations in relative terms are minor as compared to neutrophil counts and CRP. In post-operative patients with proven bacterial infections, however, HNL concentrations were further increased in contrast to neutrophil counts and CRP. Thus, the use of HNL measurements for the identification of post-operative bacterial infections seems to be superior. In an additional study on patients subjected to open-heart surgery, HNL did not show this distinction (23). This finding might relate to the new knowledge that HNL is increased in blood in patients suffering from a post-operative acute kidney injury (10). The development of acute kidney injury is common in open-heart surgery, and in these cases the monitoring of HNL in blood and/or urine has been shown to be possible to use as an early sign of kidney injury (9,10).

## The assay of HNL in serum or plasma?

HNL can be measured in any body fluid, and in blood it may be measured in serum or EDTA-plasma. Clinical studies on acute infections indicate that serum measurements are superior to plasma measurements. Serum measurements, however, require strict standardization of the blood sampling procedure in order to avoid falsely low or high concentrations. This is because neutrophils in the test tube *ex vivo* continue to release HNL, and this process is time- and temperature-dependent. A standardized procedure is easy to accomplish and requires a watch and an ambient temperature of approximately 22 °C. Thus, the blood tube is placed on the bench for 120 min ±15 min, after which the blood is centrifuged and serum carefully harvested. Preferably, vacuum tubes containing gel separators should be used.

**Figure 3.**
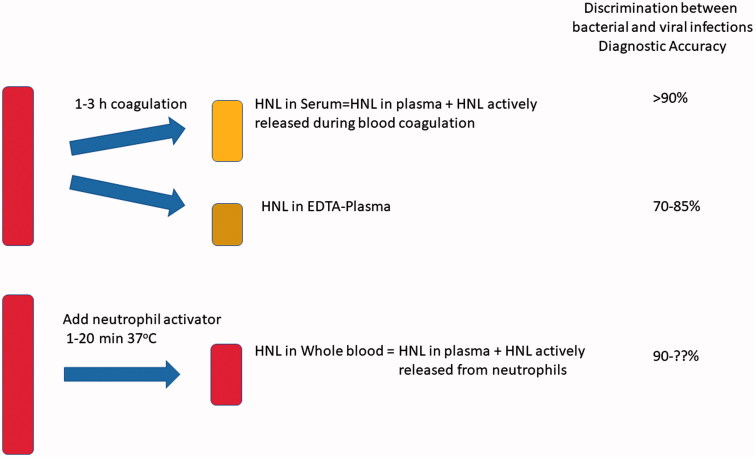
Results of measuring HNL in serum, plasma, or after whole-blood activation. The diagnostic accuracy as defined by specificity + sensitivity/2 is given for the different measurements.

Why is serum measurement of HNL superior to plasma measurement in the distinction between bacterial and viral infections? An answer to this question is illustrated in Figure 3. When measured in EDTA plasma, HNL estimates accurately reflect the circulating concentrations. These concentrations are consequences of an increased production and release of HNL from various sources in the body. However, the circulating HNL is also subject to elimination. Thus, the prevailing HNL concentrations are the results of production and elimination. If production dominates we expect increasing concentrations, whereas in processes with an increased elimination these increasing concentrations will be counteracted and result in seemingly normal or even lower concentrations of HNL. Even if almost nothing is known about the process involved in turnover of HNL in humans, we may assume that this is increased in states of inflammation. When measured in serum, elimination of HNL is obviously turned off, whereas production and release from blood cells, i.e. neutrophils, may continue. In the distinction between bacterial and viral infections it is the activity of circulating neutrophils that seems to determine the diagnostic power. Therefore, assays to accomplish this distinction should aim at determining this activity. As shown by the examples given above, the distinction is clear if blood is handled accurately and according to the instructions.

## Plasma HNL in sepsis

HNL was measured in EDTA plasma by our radioimmunoassay in 138 patients admitted to the intensive care unit with the suspicion of sepsis. HNL showed a close relationship to documented bacterial infection in patients with systemic inflammatory response syndrome (SIRS) and sepsis. In patients with SIRS the capacity of HNL to detect bacterial infection was compared to that of CRP and procalcitonin (PCT). Neither CRP nor PCT showed any capacity in this regard, whereas HNL showed an area under a ROC curve of 0.82 and with sensitivities and specificities close to 80%. Thus, plasma HNL seemed superior to the two other biomarkers. In a logistic regression analysis the odds ratio for sepsis was 23.1 for the highest concentrations of HNL as compared to 17.3 and 12.0 for the highest concentrations of PCT and CRP, respectively. These differences in odds ratios were found irrespective of whether the model was adjusted for kidney function or not. It was concluded that plasma HNL is a promising marker of sepsis in critically ill patients, since it effectively distinguished infective from non-infective causes of SIRS. The observations further suggested that the association between elevated HNL and sepsis is independent of the level of kidney (dys)function. Although HNL outperformed PCT as a sepsis marker, its role in aiding antibiotic stewardship still has to be determined. Assays that specifically detect the neutrophil-specific form of HNL in plasma or serum should also be evaluated in future sepsis studies.

## Remarks on plasma/serum measurement of HNL

In several studies the assay of HNL in serum was shown to be superior to any other means to distinguish between the two major causes of infection, i.e. bacteria or virus. This distinction is very important in the judgment of whether the patients should be prescribed antibiotics or not. In this decision the assay of HNL should be a complement to the clinical findings and a support to the doctor, since the aim should be to avoid the prescription of antibiotics to those patients who do not benefit from such intervention. Thus, an assay that with high likelihood can rule out a possible bacterial infection should have a great impact on health care and be important in the combat of the fast-growing epidemics of antibiotic resistance. At present only laboratory assays of HNL in the format of ELISA are commercially available. Such assays have a typical response time of 2–3 h and are therefore useful in the evaluation of infections in hospitalized patients or in those patients seeking care at the emergency care unit in a hospital with access to a modern laboratory. In the primary care and in most emergency care units a rapid point-of-care assay would be desirable.

## Whole-blood assay of HNL with a rapid point-of-care assay

**Figure 4.**
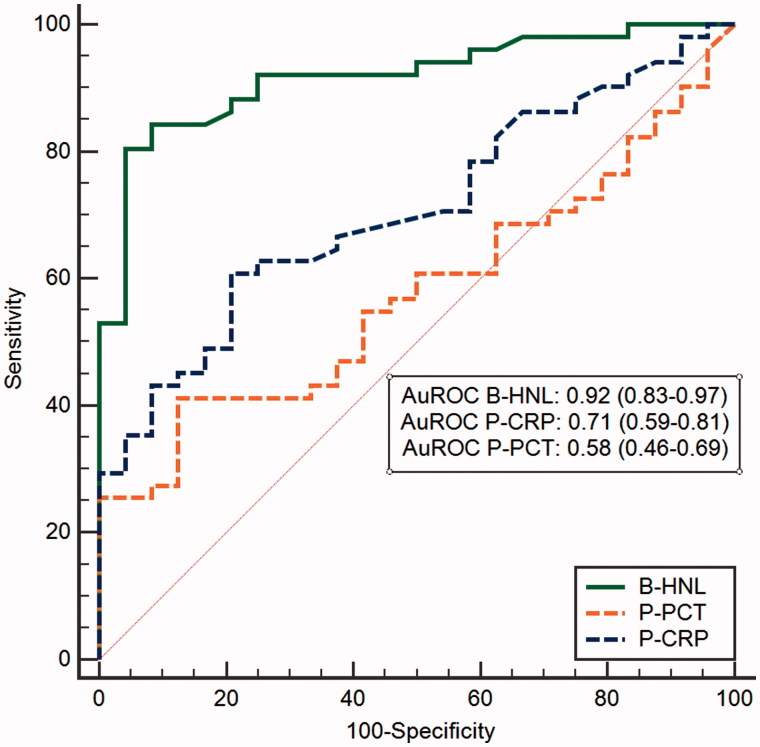
A ROC curve analysis of HNL after whole-blood activation in comparison with plasma concentrations of CRP and procalcitonin. Patients had symptoms of respiratory infections, and their infections were objectively verified by microbiological testing. The AuROCs are given in the figure.

One of the major obstacles and causes of variation in serum HNL results is the pre-analytical handling of the blood. As pointed out above, a standardized procedure in this regard is mandatory and very often difficult to achieve in a busy clinical setting. We have therefore sought other solutions that might eliminate these problems. One requirement to an alternative procedure was that the results of such a new user-independent procedure should reflect what is achieved with a coagulation-dependent activation of neutrophils. The procedure described below fulfills this requirement, although the results are achieved within 5–10 min after blood draw. The idea behind the invention was that the activation of neutrophils in whole blood by a suitable neutrophil activator should induce the extracellular release of HNL and that the extent of the release would reflect the state of activation of the neutrophil and mimic what was achieved in terms of HNL release after 2 h blood coagulation. The chosen activator was the tripeptide fMLP. Initial experiments with blood neutrophils isolated from more than 100 patients with infections and non-infected controls showed that the extent of release of HNL from the purified neutrophils after fMLP exposure was closely correlated to the serum concentrations of the same subjects. This was a very important observation, since it clearly showed that the serum concentrations achieved after blood coagulation mainly are dependent on the activity of the neutrophils and less on the numbers of cells. Based on such results we conducted a large clinical study (Bio-X) on about 750 infected and non-infected subjects (24–26). Whole blood was obtained and incubated for 20 min with fMLP, after which HNL was measured by a lateral-flow point-of-care assay. The results were compared to CRP, PCT, and the expression of CD64 on neutrophil surface and showed that measurements of HNL in activated whole blood were clearly superior to any of the other three biomarkers. For the whole group of patients the area under the ROC curve was 0.91 for HNL and 0.70 and 0.63 for CD64 and PCT, respectively. Obviously, HNL was superior to these biomarkers in most infectious diseases (Table 4). CRP was not included in these calculations, since this biomarker had been known to the adjudicators. However, when we studied patients with respiratory infections separately we included CRP in the calculations together with CD64 and PCT, and a very interesting pattern emerged. CRP and HNL had a similar diagnostic capacity when the distinction was based on clinical observations only. However, when the diagnosis was confirmed by objective microbiological tests the diagnostic power of HNL was increased from AuROC 0.82 to 0.92, whereas AuROC for CRP was decreased from 0.82 to 0.74 (Figure 4). The same pattern was repeated whether upper respiratory or lower respiratory infections were calculated separately or whether the results were calculated on the basis of symptoms of respiratory infections such as cough, stuffy nose, or sore throat. These results tell us two things. One is that the use of CRP overestimates bacteria as the cause of the infection, and secondly, and more importantly, that the measurement of HNL in whole blood after activation specifically reflects the body’s response to a bacterial infection.

**Figure:**
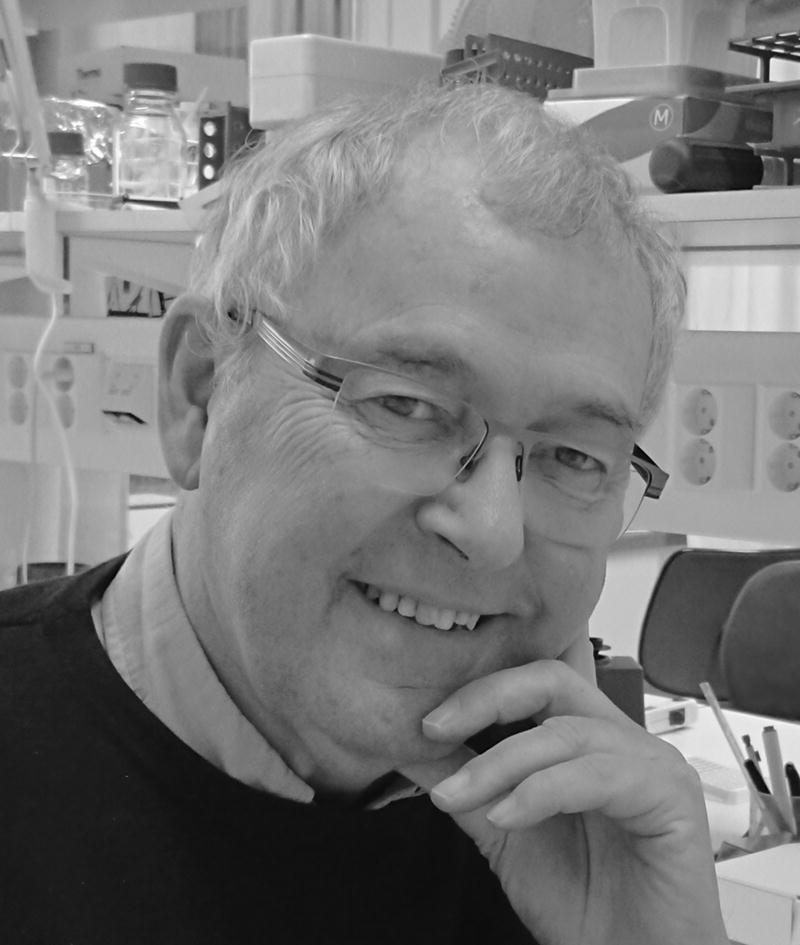
Per Venge and his collaborators have been awarded the €1 million EU prize, the ‘*Horizon Prize for better use of antibiotics*’. This review summarizes how their combined research efforts have led to a unique solution to reliably detect bacterial infections.

**Table 4.** Distinction between bacterial and viral causes of various infections as expressed by areas under ROC curves (AuROC).

Diagnosis	B-HNL AuROC (95% CI)	CD64 AuROC (95% CI)	PCT AuROC (95% CI)
Bacterial pneumonia	0.868 (0.771–0.935)	0.641 (0.523–0.748)***	0.647 (0.529–0.753)***
Streptococcal tonsillitis	0.905 (0.815–0.960)	0.765 (0.653–0.855)*	0.537 (0.418–0.653)***
Urinary tract infection	0.899 (0.805–0.957)	0.707 (0.588–0.808)**	0.808 (0.698–0.891)
Bacterial GI infection	0.773 (0.635–0.887)	0.832 (0.703–0.921)	0.698 (0.555–0.817)
Erysipelas	0.951 (0.856–0.991)	0.683 (0.544–0.802)**	0.676 (0.534–0.991)**
Sepsis, endocarditis	0.980 (0.899–0.999)	0.824 (0.697–0.914)	0.938 (0.838–0.986)
URTI (excluding tonsillitis)	0.944 (0.838–0.990)	0.677 (0.544–0.792)***	0.515 (0.383–0.645)***

*p < 0.05, **p < 0.01, ***p < 0.001.

URTI: upper respiratory tract infection.

In more recent studies we showed that whole-blood activation and release of HNL may be optimal after 3–5 min, i.e. considerably shorter than 20 min, which was the time used in the Bio-X study (24–26). Based on such experiments, point-of-care assays are under development in which activation of whole blood and measurements of HNL are performed simultaneously in the small handheld instrument. Feasibility experiments showed that this is possible and will allow the results to be obtained within 5–10 min after blood draw. Thus, the fully developed assays will allow for a rapid point-of-care evaluation of acute infections with a high degree of diagnostic accuracy and will support the physician in the decision of prescribing antibiotics or not.

## Concluding remarks

The early and accurate discrimination between bacterial or viral causes of acute infections is key to the better use of antibiotics and will help slow down the fast-growing resistance to commonly used antibiotics. This discrimination is in the vast majority of cases possible to achieve by the blood assay of the biomarker human neutrophil lipocalin (HNL), which we showed to be uniquely increased in patients suffering from bacterial infections. The development of a robust and easy to use point-of-care assay of HNL, which is fast and accurate, may be a major breakthrough in the management of patients with acute infections and help reduce the misuse of antibiotics.
